# Professional Ethics and Honor

**Published:** 1890-04

**Authors:** W. Storer How

**Affiliations:** Philadelphia, Pa.


### Professional Ethics and Honor.

BY W. STORER HOW, D.D.S., PHILADELPHIA, PA.

Read before the Mississippi Valley Dental Association, at Cincinnati, Ohio, March, 1890.

A proposition that the subject of ethics should be ethically discussed would seem to be so manifestly self-evident that assent should follow its mere statement, but a moment's consideration brings to mind the fact that whatever the profession of a person may be, the reduction of professed ethics to practice is a matter of such confessedly great difficulty, not to say rarity, that the discussion of the subject is an undertaking of extreme delicacy and intricacy, not unmixed with danger of personal conflict.

The hand of history points with awful significance to the blood stains on its pages caused by violent discussions of questions of an ethical nature, and the most sanguinary combats have often been between those parties professing peculiar regard for principles of personal purity and righteousness.

The advent of the Prince of Peace, and the gradual growth of real rightousness from that day to this has, however, made it at least probably practicable for men now to meet and compare their views on subjects of correct conduct without imminent risk of coming to blows in the event of disagreements during the discussion.

Members of a dental society as a body of men associated for the avowed object of promoting unity of action in the prosecution of a commendable calling, and living at a time in a country wherein the largest liberty of speech and conduct prevails, are especially favored in facilities for the unrestricted consideration of every suitable subject connected with their association as representative American dentists.

Ethics as intimately related to the practice of dentistry was made the subject for discussion at the 1866 meeting of the American Dental Association at Boston, Mass., and what has been termed a "Code of Ethics," was formulated as a prescription of definite rules of professional conduct within which lines every dentist would be expected to practice in order to be eligible for, or to continue in the membership of that association. That code consists of four articles bearing titles as follows: Article I. The Duties of the Profession to their Patients. II. Maintaining Professional Character. III. The Relative Duties of Dentists and Physicians. IV. The Mutual Duties of the Profession and the Public.

The law of association and the laws of associations are not always practically in harmony, however concertive they may seem to be in theory or on paper. The disposition and the determination of men to group themselves because their individual pursuits or vocations are similar in kind or character, may be said to find an initial expression in the common desire of every person for the companionship and encouragement of some other congenial person. Hence, broadly considered, arise every species of social aggregation and organization. Hence, also, arises the necessity for the clear definition of, and a general assent to a well understood expression of the courses of conduct which may be agreeable or disagreeable to the members of the group.

In the present instance the code has, for more than twenty years, served as a working formula for the generally harmonious membership of that association which has been in a great degree representative of the steadily advancing dental profession in America.

At a time and in a land wherein the actual practice of ethical precepts has become more general than in any previous age or other country, it is certainly pertinent to the declared objects of such associations that every reasonable endeavor should be made to have professional practice conform to professional ethics at every proper point of the published agreement.

With a view to the careful consideration of some fundamental features of the code and without prejudice to the features not commented upon, the 1st Section of Article 2 is here quoted :

" A member of the dental profession is bound to maintain its honor, and to labor earnestly to extend its sphere of usefulness. He should avoid everything in language and conduct calculated to dishonor his profession, and should ever manifest a due respect for his brethren. The young should show special respect to their seniors ; the aged, special encouragement to their juniors."

*Honor* is a word of so many meanings that the sense in which it is used in Section 1 may be open to question, or at least to a clear definition. According to Worcester, the prime significance of honor is : " Esteem or regard founded on worth or opinion ; reputation ; repute ; fame ; glory."

" Honor makes a great part of the reward of all honorable professions."—*Adam Smith.*

> " Honor and shame from no condition rise;
> Act well your part—there all the honor lies."—*Pope.*

Webster defines it as primarily : " Esteem due or paid to worth ; high estimation ; consideration."

" A nice sense of what is right, just, and true, with a course of life correspondent thereto."

> " Say, what is honor. 'Tis the finest sense
> Of justice which the human mind can frame,
> Intent each lurking fraility to disclaim,
> And guard the way of life from all offense
> Suffered or done."—*Wordsworth.*

Of these few from the many significances of the word, we may reasonably consider this last citation to embody the ethical meaning of honor as applicable to a profession, and as including its practice in a course of life corresponding thereto.

It will be noticed that the crowning quality of honor is justice in its finest sense. Webster says that "justice is the quality of being just, the rendering to every one his due, right, or desert; practical conformity to the laws, and to principles of rectitude in the dealings of men with each other; honesty; integrity in commerce or mutual intercourse; strict conformity to right and obligation; rectitude; integrity; impartiality."

When, therefore, we think or speak of professional honor it is evident that we should have in mind some of the noblest attributes of man. Because nothing is more certain than the fact that, since the professional honor as a whole is the aggregate of the honor of its members as individuals, so the quality of that honor as put in practice will indicate surely the real estimation and significance in which the word is held by a representative member.

The code correctly counts upon the strictly representative professional character of every member, and it is therefore of the first consequence that his conception of what it is to maintain its honor, should be of the highest and noblest kind. The subject is of the more importance, because of the prevalence of confused and even perverted notions, as to what is actually expressed and implied in the common usage respecting honor. Indeed, it is often of great assistance in discovering the real import of a word to first find out what it clearly is not.

Professional honor is not akin to pride, which is "inordinate self-esteem; behavior which indicates contempt, or slight esteem of others." "Pride is that exalted idea of our state, qualifications, or attainments which exceeds the boundaries of justice, and induces us to look down upon supposed inferiors with some degree of unmerited contempt."—*Cogan.* The sharp contrast between this which "exceeds the boundaries of justice" and "the finest sense of justice the human mind can frame," sufficiently discriminates words which too often are used as if they were ethically of the same or like quality. It is therefore clearly incompatible with professional honor to "look down upon supposed inferiors with some degree of unmerited contempt." The contrast also enables us to see distinctly that honor, especially professional, is not self-esteem; not egotistic, but altruistic —it is what is thought of others—what others think of the professor, in the present case, of the dentist—of you and of me. "It is esteem due to worth; a nice sense of what is right, just and true with a course of life consequent thereto." In other words, a professional man is to be manifestly right, just and true, that due honor will be paid him by all, and especially by his brethren who are capable of the esteem due to worth.

The section of the dental code of ethics under consideration has special reference to the relation of dentists to each other as associated members of the dental profession. It is certainly permissible, if not essential, to an intellegent discussion of these relations to inquire into the nature of a profession as distinguishable from other associations or callings.

At the outset of this contemplation it was observed that associations or societies are composed of agreeing aggregations or persons engaged in similar pursuits, and notice was taken of the fact that the present circumstances and conditions of such associations differ from those of the past. To-day it is not proper in a republic for one group or class to assume inherent superiority over another group or class. Nor is it just and honorable for one class to endeavor to magnify its own importance by contemptuously decrying another class.

The words superior and inferior have in this country lost their old time meanings as indicating respectively dominant and servile classes. Here the professional man as a man stands on the same legal level with every other worthy workman. The word work has steadily risen in appreciation until here and now men generally are no longer ashamed of being regularly at work. In fact, the laudable ambition of every truly American youth is to prepare for and enter upon a field of honest work.

In earlier times professional work or service was compensated by presents, the amount of which in current value was determined by a benificiary who gave whatever he might graciously be pleased to bestow. Subsequently the fee or "honorarium" came to have a somewhat definite value for a single service, or for a successful series of services. "It is still customary in Great Britain to estimate professional fees, honoraria of all kinds, complimentary subscriptions, prices of pictures, etc., in guineas; to give a physician three sovereigns and three shillings rather than three sovereigns alone, or even three sovereigns and five shillings, supposed to make the transaction differ from a mere mercantile one, and thus to veil the sordidness which is fancied to attach to pounds, shillings and pence."

There is instructive significance in the fee of a guinea that has a value of one shilling more than a pound, and the extra is given to show that a shilling more or less is of no consequence to the aristocratic donor, who has a lordly disdain of money, which is valued in small sums only by inferior or servile persons—such as the physician and others of his kind. There are also people in this country who affect a similar contempt for those whose services are compensated with money. Those aristocrats superciliously look down upon professional men, as some of these in turn look down on persons whose services receive a smaller reward, and apishly "put on airs" as a sort of exclusive professional atmosphere.

The implication of the guinea honorarium that the shilling tip will be received without a qualm is certainly not flattering to the presumptive self-respect of the professional man. Let us by all praiseworthy means cultivate in the youngest of the professions a sense of honor sufficiently sensitive to recoil from the degradation of taking a superciliously bestowed gratuity in lieu of an equitable cash equivalent for honestly rendered professional services.

It should ever be borne in mind that the true consideration, discussion and elucidation of professional ethics in general, or in particular, presupposes the personal possession and practice of the finest sense of honor associated with a courtesy of demeanor which, while delicate in demonstration, is yet determined in defense of the real rights of the humblest among true men in every honest vocation.

The careful cultivation of a nice sense of what is right, just, and true, with a correspondent course of life, will surely secure for the exemplar the esteem paid to worth as a great part of the reward of all honorable professions or vocations.

Sometime after the completion of the preceding paper the writer finds in the New York *Independent* of February 20, 1890, an article by Prof. W. G. Sumner, of Yale University, from which some pertinent extracts are here appended.

"* * * An American will be sure to be astonished on the continent of Europe by the scruples and mannerisms with which professional men surround the acceptance of the remuneration which they are quite as eager to get as any Yankee. It looks as if they were ashamed of their livelihood, or felt themselves lowered by taking what they have fairly earned. It is not worth while to seek such evidence of the remnants of the same sentiments as one could find among ourselves.

"The feudal period produced a new and still more intense development of the same sentiment in a somewhat changed form. All the industrial forms of livelihood were regarded as servile in comparison with the functions of the fighting classes and their ecclesiastical allies. The learned class were on the line between, unless they sought ecclesiastical rank, or, later, as legists, made themselves independently necessary.

"It is only very slowly that the notions of an industrial and commercial civilization have fought their way during the last five hundred years against the militant notions. The latter have had, and still largely retain, the aroma of aristocracy. Therefore they are affected by many who do not understand them. The dictum that labor is noble, or dignified, has been a watchword of industrialism in its struggle to assert itself against militancy; but the industrial classes, as fast as they have attained wealth, have deserted industrialism to seek alliance with aristocracy, or to adopt the modes of life which the militant tradition marks as more honorable.

"* * * What, then, shall we infer? Is the sweet doctrine that labor is dignified and ennobling all wrong? Were the ancients right? Is labor for pay always degrading, and does it become worse and worse as we go down the grades from those occupations which have the most brain work and least manual work to those which have the most manual work and least brain work? The issue is clear and it is not difficult. It would do great good to solve it completely for it would clear up our ideas on many topics which are at present in confusion.

"I maintain that labor has no moral quality at all. Every function in social work which is useful to society is just as meritorious in every way as any other, each being suitable and an object of choice to the person who performs it. The moral quality depends on the way in which it is performed. The social estimate and the personal worth which should be ascribed to social functions depend on the way in which the man we have in mind does his duty. It is not capable of generalization, and there is no reason for generalizing it.

"The educational value of different social functions is equal, and the degree of human perfection which can be got out of them is equal. It developes a man in all moral excellence, and in all that vague 'elevation' which plays such a prominent part in social speculation just as much to be a good and faithful hod-carrier as to be a good and faithful statesman."

It follows from the foregoing that current discussions of questions concerning the ethics and honor of both the medical and dental professions might well be modified by more modern views of the true relations of the several departments of reputable labor, and the correlative organizations for improvement in the methods of practice. These will doubtless be best promoted by a due recognition and just estimation of the rights and services of the individual workman in every praiseworthy calling.

The true incentive to excellence and faithfulness in any useful occupation is not to be found in the fostering of a feeling of social superiority, as of an archæologist over an artisan; an astronomer over an artist; a doctor over a dentist; an embassador over an electrician; an entomologist over an editor; a lawyer over a laborer; a minister over a mechanic; a philosopher over a photographer; a physician over a pharmacist; a scientist over a sailor.

These sociologically absurd antitheses are adduced to add emphasis to the conclusion that professional conduct is consonant with true ethics and honor only when not contaminated by pride or contempt in the treatment of trustworthy toilers in other lines of lucrative labor.

